# Kidney Biopsy Un-masquerading Plasma Cell Leukemia Diagnosed as Acute Myeloid Leukemia: An Unusual Clinical Experience

**DOI:** 10.7759/cureus.42909

**Published:** 2023-08-03

**Authors:** Abdul Hannan A Rasheed, Xin Zhang, Song Liu, Maria M Picken, Kavitha Vellanki

**Affiliations:** 1 Medicine/Nephrology, Loyola University Medical Center, Maywood, USA; 2 Pathology, Loyola University Medical Center, Maywood, USA; 3 Medicine/Nephrology, Edward Hines, Jr. VA Hospital, Hines, USA

**Keywords:** plasma cell leukemia, rare disorder, acute myeloid leukemia, overlapping hematological malignancies, nephrotic-range proteinuria, acute kidney injury, hypercalcemia

## Abstract

Plasma cell leukemia (PCL) is a rare aggressive variant of plasma cell myeloma. The differential diagnosis of PCL includes multiple myeloma (MM), other leukemias, and lymphomas with abnormal cells circulating in the peripheral blood. In addition, infectious or autoimmune diseases can cause reactive polyclonal plasmacytosis, which could confuse us with PCL occasionally. Sometimes, blastoid morphology can cause confusion in diagnosis, and immunohistochemistry is needed to differentiate PCL from other forms of leukemias and lymphomas. Here, we present a rare case of PCL diagnosed as acute myeloid leukemia (AML) with kidney biopsy establishing the correct diagnosis.

## Introduction

Plasma cell leukemia (PCL) is a rare and aggressive form of multiple myeloma (MM) classified into primary if it occurs de novo and secondary from the leukemic transformation of MM. The clinical presentation can be like MM. Diagnosis is confirmed by ≥ 5% plasma cells on a conventional peripheral blood smear (not on flow cytometry) and a monoclonal population of plasma cells on bone marrow (BM) biopsy [1-2]. We present an unusual clinical experience where peripheral smear and BM biopsy could be misleading and a kidney biopsy helped in establishing the right hematological diagnosis.

This article was previously presented as a meeting abstract at the ASN Kidney Week in Orlando, FL, on November 4, 2020.

## Case presentation

A 64-year-old female, with a history of hypertension, presented with progressive weakness and fatigue for two weeks. Her blood pressure was 127/81 mmHg, pulse rate of 74/min, respiratory rate of 16/min, and temperature of 98.5 °F. The rest of the examination was unremarkable. Initial investigations showed anemia, leukocytosis, thrombocytopenia, acute kidney injury (AKI), with a creatinine of 6.76 mg/dL compared to baseline of 1.2 mg/dL, and severe hypercalcemia with a corrected calcium of 14.9 mg/dL (Table 1).

**Table 1:** Investigations at admission, at day 21 (dialysis initiation), and four months after admission (off dialysis for six weeks) WBC (White blood count), Hgb (Hemoglobin), BUN (Blood urea nitrogen)

Component	At Admission	At Day 21	At 4 Months	Reference Range and Units
WBC	16.4	9.8	4.5	3.5–10.5 x 10^9 ^/L
Hgb	7.5	8.1	7.8	11.5–15.5 gm/dL
Platelets	133	137	37	150–400 x 10^9 ^/L
Sodium	139	143	139	136–144 mmol/L
Potassium	4.3	5.6	4.6	3.3–5.1 mmol/L
Chloride	95	111	105	98–108 mmol/L
CO2	29	13	20	20–32 mmol/L
Anion Gap	15	19	14	4–16
BUN	44	62	45	7–22 mg/dL
Creatinine	6.76	10.23	2.9	0.6–1.4 mg/dL
Glucose	107	92	134	70–100 mg/dL
Calcium	14.5	8.9	8.3	8.9–10.3 mg/dL

**Figure 1:**
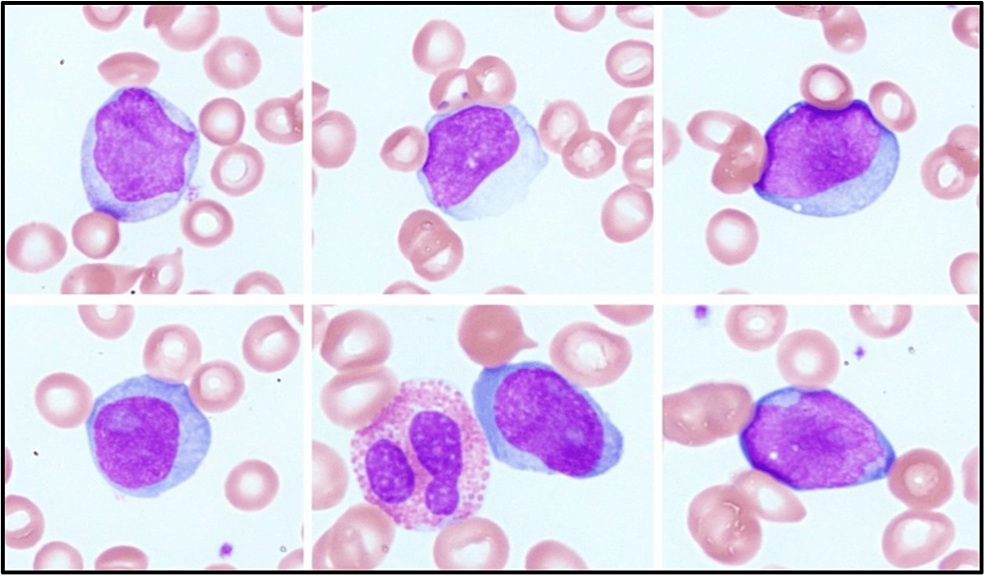
Peripheral blood smear with Wright stain Circulating cells with blastoid features demonstrating large cells, open chromatin, moderate basophilic cytoplasm, and prominent nucleoli

Peripheral blood smear on presentation revealed 12% blastoid cells (Figure 1), and preliminary diagnosis of acute leukemia was considered with myeloma less likely as immunoglobulins were within normal limits.

Urine analysis showed 3+ protein with no hematuria, and the urine spot protein/creatinine ratio was 11.7 g/g. A nephrology service was consulted for further management of AKI and hypercalcemia. Serum free light chains, serum, and urine electrophoresis with immunofixation were ordered for workup. A BM biopsy showed 74% blasts, and flow cytometry was positive for myeloid markers, including CD33, CD38, CD45, and cMPO, and cytogenetic studies showed loss of chromosome 8, loss of core-binding factor, beta subunit (CBFB) on chromosome 16, loss of B cell receptor (BCR) on chromosome 22, loss of tumor protein 53 (TP53), and low-level loss of chromosome 13. A diagnosis of acute myeloid leukemia (AML) with blasts was made, and chemotherapy consisting of azacytidine and venetoclax was initiated on day nine of her presentation, held after the first dose due to the development of upper gastrointestinal bleeding that required endoscopic interventions. Her paraproteinemia workup that was initiated on presentation subsequently revealed significantly elevated serum free lambda light chains, and serum protein electrophoresis showed an elevated concentration of alpha 2 globulin (Table 2), with immunofixation showing a restricted band of free lambda light chains in the alpha 2 regions.

**Table 2:** Serum free light chains and protein electrophoresis K/L ratio (Kappa/Lambda ratio)

Component	Results	Reference Range and Units
Free kappa light chain	145.1	3.3–19.4 mg/L
Free lambda light chain	27,360.7	5.7–26.3 mg/L
Free K/L ratio	0.01	0.26–1.65
Protein	7.0	6.2–8.0 mg/dL
Albumin	3.6	3.7–4.8 mg/dL
Alpha 1	0.2	0.1–0.2 mg/dL
Alpha 2	1.4	0.6–1.0 mg/dL
Beta	0.7	0.6–1.1 mg/dL
Interpretation	Atypical band in the alpha 2 region	

**Figure 2:**
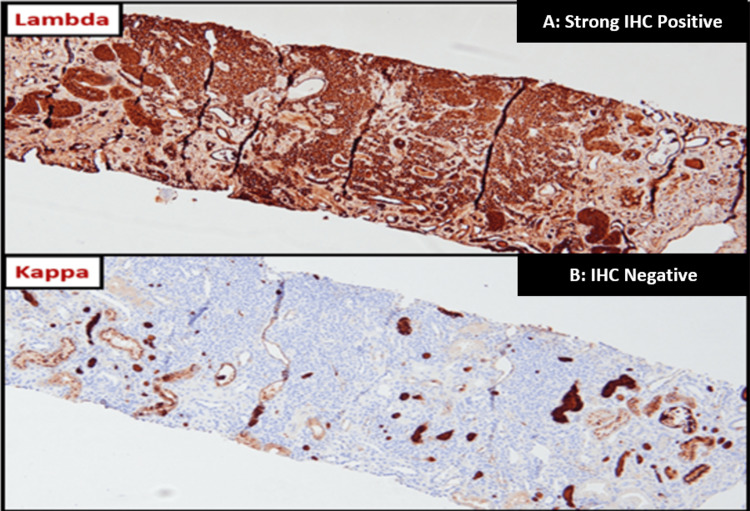
Kidney biopsy with CD138 immunohistochemistry (IHC) stain

While the possibility of two primary processes was initially contemplated, since the co-existence of AML and paraproteinemia is extremely unusual [2], a kidney biopsy for definitive diagnosis was proposed by nephrology service, but delayed due to thrombocytopenia and overall unstable clinical condition. She eventually did receive a kidney biopsy two weeks into her initial presentation, with AML being considered the primary process during this time as a BM biopsy did not reveal plasma cells (misidentified as myeloid blasts). A preliminary kidney report revealed lambda light chain cast nephropathy. On further staining of the kidney biopsy samples for CD138 on immunohistochemistry (IHC), it did reveal infiltration with plasma cells with strong positivity for lambda but not kappa light chains (Figure 2).

Congo red stain was negative. A BM biopsy was repeated after the preliminary kidney biopsy findings, and a re-examination of peripheral blood smear and BM biopsy with CD138 IHC staining confirmed that the blastoid-like cells with myeloid features were indeed plasma cells. Kappa lambda in situ hybridization revealed lambda light chain restriction, confirming the diagnosis of PCL. This not only led to a change in her diagnosis but prompt targeted treatment for PCL was initiated. She received three sessions of plasma exchange and induction chemotherapy with dexamethasone, cyclophosphamide, carfilzomib, lenalidomide, and daratumumab. Her hospital course was complicated with hypotension from sepsis and gastrointestinal bleeding, with renal function declining after initial improvement with the management of hypercalcemia. She ultimately required renal replacement therapy on day 21 of her admission. Her renal function eventually recovered, and dialysis was held after 10 weeks of dialysis initiation. She had residual stage 4 CKD with new baseline creatinine of 2.8-2.9 mg/dl (Table 1). After five cycles of chemotherapy, she eventually received a hematopoietic stem cell transplant after eight months of presentation and remained dialysis independent.

## Discussion

PCL is a rare aggressive form of MM with a dismal prognosis despite advancements in cancer treatment in general. As per the recently updated International Myeloma Working Group consensus definition in December 2021, the diagnosis of primary PCL is defined by the presence of 5% or more plasma cells in peripheral blood smear (used to be ≥ 20% previously) in patients otherwise diagnosed with symptomatic MM [1]. The prior reported incidence of four cases per 10,000,000 persons per year across the United States and Europe [3] is expected to be higher with the newer definition [1]. Clinical presentation of PCL is like myeloma (AKI, hypercalcemia, anemia, and lytic bone lesions) [4], and it can have overlapping features of other leukemias (leukocytosis, splenomegaly, hepatomegaly, thrombocytopenia, and anemia). The morphological features of plasma cells on peripheral blood smear can differ depending on their maturity and can mimic myeloblasts in rare instances [5-8]. In such cases, IHC with plasma cell/myeloma marker CD 138 can help differentiate plasma cells from myeloblasts. The overall prognosis remains poor despite the use of high-dose chemotherapy with autologous hematopoietic transplantation. The median overall survival was reported to be 12-46 months depending on risk factors on presentation [9].

While AKI can be a presenting feature, it is extremely unusual to require a kidney biopsy for a definitive diagnosis [10]. Our experience is the first case to be reported where a kidney biopsy unmasked the diagnosis of PCL when the original diagnosis of AML was made based on peripheral smear and BM biopsy findings. There were several misleading features suggesting AML (leukocytosis, splenomegaly, myeloblastic features of circulating plasma cells, and positive myeloid markers on flow cytometry, as shown in Table 3).

**Table 3:** Overlapping features of AML and plasmacytoma in our case AKI (Acute kidney injury), FISH (Fluorescence in situ hybridization), CBFB (Core binding factor, beta subunit), BCR (B-Cell receptor), TP53 (Tumor protein 53)

Characteristics	Findings in Our Patient	
Physical findings	Splenomegaly.	
Initial investigations	Leukocytosis with peripheral blasts; hypercalcemia; AKI.	
Flow cytometry	Positive for CD 33, CD 117, CD 56, CD 38, and MPO; negative for CD19, CD20, surface kappa and lambda light chains, CD3, TDT, CD4, CD8, CD34, CD2, CD7, CD15, HLA-DR, CD117, CD11C, CD14, CD64, and CD22. CD 138 was not done on initial flow cytometry.	
		Cytogenetics - FISH	Loss of RARA (chromosome 17). AML profile: loss of chromosome 8, loss of CBFB (chromosome 16), and loss of BCR (chromosome 22). Myeloma profile: loss of TP53, 1ow level loss of chromosome 13.	

Several reports of AML presenting with hypercalcemia have been published in the literature too, but the exact mechanism has yet to be elucidated [11-12]. In our patient, hypercalcemia was initially attributed to bone osteoclastic activity from AML. Similarly, AML presenting with nephrotic syndrome is rare but has been reported in the literature [13]. Nephrology-initiated workup for AKI, hypercalcemia, and proteinuria on presentation revealed severely elevated serum free lambda light chains with serum immunofixation results in line with the free light chain results. Preliminary kidney biopsy findings of lambda light chain cast nephropathy were what lead our haemato-oncologists to revisit the peripheral smear and BM biopsy findings and further staining with CD 138 of the kidney, and repeat BM biopsies confirmed the diagnosis of PCL. It is important to recognize that PCL is rare, and PCL mimicking AML is even rarer.

## Conclusions

PCL with blastoid morphology can pose a diagnostic challenge by mimicking acute leukemia. Our experience illustrates the importance of the emerging field of onco-nephrology and the need for a collaborative approach with hematologists, pathologists, and oncologists in providing care to such patients with complex clinical presentations. We hope our experience raises awareness of plasma cells mimicking myeloblasts in the nephrology community and the importance of IHC staining with CD 138 in differentiating one from the other. Prompt identification and diagnosis are paramount to start effective treatment in PCL that has an ominous prognosis.
